# Texercise: The Evolution of a Health Promotion Program

**DOI:** 10.3389/fpubh.2014.00262

**Published:** 2015-04-27

**Authors:** Holly Riley

**Affiliations:** ^1^Center for Consumer and External Affairs, Texas Department of Aging and Disbality Services, Austin, TX, USA

**Keywords:** health promotions, older adults, Texas, physical activity and wellbeing, partnership, history

Texercise, a program developed by the state of Texas, promotes healthy lifestyle choices to help residents age and live their best. This commentary provides the conceptualization of the program, its growth over the years and how it is disseminated. The current federal direction on evidence-based prevention program for seniors from the Administration for Community Living and its potential influences on the Texercise program are also provided.

## Conceptualization

Texercise, a health promotion program of the Texas Department of Aging and Disability Services (DADS), has seen dramatic growth since its inception in 1998. Originally developed by the state unit on aging (SUA) to support the Aging Texas Well initiative (1), Texercise is now a comprehensive health program with outreach and programing across the state. Texas’ obesity statistics and health indicators (2) highlight the need to help people improve their health and quality of life. The Aging Texas Well initiative (3) focuses on preparing Texas for a growing aging population and helps Texans understand the importance of planning for their futures. Good physical health is a key component of this initiative.

When first conceived, Texercise was an 8-month long statewide exercise campaign created to support the Aging Texas Well campaign. The primary Aging Texas Well message was that individuals, local communities and the state need to take the appropriate steps to prepare for an aging society. Texercise was developed to support that message, focusing on physical health and wellness action steps. After the initial Texercise campaign, agency leaders saw the value of a permanent state-level health promotions program that would support the work of Texas’ 28 Area Agencies on Aging (AAA) and the SUA’s activities.

The AAAs saw the potential to use Texercise in implementing Title IIID of the Older Americans Act, which focuses on disease prevention and health promotion. And the SUA saw the potential to enhance its messaging to reach beyond the 60-plus population into worksites and the community. Texercise also offered the SUA opportunities to involve state-level programs and business in partnerships through the program.

## Dissemination

After the launch, a small Texercise booklet featuring physical activity exercises and recommendations was developed with the guidance of Dr. Kenneth H. Cooper of the Cooper Aerobics Institute. The initial reaction to the booklet was overwhelming. The aging network, state partners, and older adults requested more, free wellness resources featuring practical information. The positive feedback from these stakeholders helped to generate a more comprehensive Texercise handbook, website, and fitness fact sheets.

At this point, the primary distribution methods for Texercise resources were the states 28 AAAs and a handful of state-level partners. Partnerships were developed with the Texas Governor’s Advisory Council on Physical Fitness, major non-profit associations and statewide media organizations to increase awareness of Texercise. As organizations outside the aging network learned about the program, they began requesting Texercise resources and tools. Simultaneously, non-traditional partnerships (e.g., trade associations, civic groups, faith-based entities, private industry, and businesses) were developed to share Texercise resources.

In 2004, the Texas Legislature reorganized Texas’ Health and Human Services agencies. The SUA, then known as the Texas Department of Aging, became the Texas DADS (4). The creation of DADS expanded the Texercise program’s primary population (60+) to include people 45 and over as well as people with intellectual and developmental disabilities. It also provided opportunities to further the program’s resources and delivery methods. New partnerships were developed, including partners that provide in-kind incentives and event development.

*Texercise Classic*, a 12-week program, was developed to accommodate requests from community organizations wanting a group exercise program. *Texercise Classic* includes motivational and recognition resources along with the Texercise handbook. A 30-min exercise DVD, featuring balance, strength, endurance, and flexibility exercises, was created to illustrate how to perform the exercises. Immediately, *Texercise Classic* was a hit with senior centers, nursing homes, assisted living facilities, faith based organizations, and worksites.

The expansion of the target population and these resources posed a welcomed challenge for the Texercise program. More staff was needed to manage the demand and growth of the program and its in-kind partnerships. In response, DADS dedicated more staff time to administer Texercise, develop partnerships and meet the needs of Texas communities.

Another challenge was keeping the program relevant and timely. Baby boomers were turning 60, and they wanted a program that represented their generation. A major redesign of Texercise in 2009 resulted in a fresh, engaging look, and updated content of all the program’s resources.

## Aligning with Evidence-Base Movement

Even with heightened awareness of the negative health outcomes associated with obesity, the United States has not seen a decline in obesity and unhealthy lifestyles. More structured, comprehensive disease prevention and wellness programs are in demand. Funders want to see returns on their investment, and many now require that their funding be spent on programs that have been proven to be effective and are evidence-based.

In 2012, the Administration on Aging began requiring that Title IIID Older Americans Act monies be spent on evidence-based programs (5). DADS leadership was committed to ensuring the aging and disability networks could continue to use the Texercise program’s resources. In 2013, DADS contracted with Texas A&M School of Public Health to develop a Texercise component that promises to achieve evidence-based recognition.

This new component (6, 7), *Texercise Select*, is the perfect complement to existing evidence-based health programs. While many of these programs focus on behavior modifications to address a specific need, *Texercise Select* emphasizes prevention through healthy behaviors. It features structured, facilitator-led classes that focus on nutrition and physical activity. Two classes for 10 weeks are administered in a group setting. The classes and associated materials provide participants with a chance to develop healthy habits while also creating a social support group. Goals and barriers are discussed in this group setting, as well as opportunities to recognize positive changes.

Department of Aging and Disability Services Texercise program had been recognized by the International Council on Active Aging and the Centers for Disease Control and Prevention’s Reference Guide of Physical Activity Programs for older adults. In addition, the President’s Council on fitness, sports and nutrition and the Texas Cardiovascular Disease and Stroke Council also have recognized the Texercise program for its community leadership. The development of *Texercise Select* is expected to increase awareness and recognition of Texercise as an evidence-based program. This alignment with the evidence-based movement will help DADS reach more people with the message that through regular preventative habits, Texans can age and live their best for many years to come.

## Conflict of Interest Statement

The author declares that the research was conducted in the absence of any commercial or financial relationships that could be construed as a potential conflict of interest.

This paper is included in the Research Topic, “Evidence-Based Programming for Older Adults.” This Research Topic received partial funding from multiple government and private organizations/agencies; however, the views, findings, and conclusions in these articles are those of the authors and do not necessarily represent the official position of these organizations/agencies. All papers published in the Research Topic received peer review from members of the Frontiers in Public Health (Public Health Education and Promotion section) panel of Review Editors. Because this Research Topic represents work closely associated with a nationwide evidence-based movement in the US, many of the authors and/or Review Editors may have worked together previously in some fashion. Review Editors were purposively selected based on their expertise with evaluation and/or evidence-based programming for older adults. Review Editors were independent of named authors on any given article published in this volume.
